# Using Capture-Recapture Methods to Estimate the Population of People Who Inject Drugs in Washington, DC

**DOI:** 10.1007/s10461-015-1085-z

**Published:** 2015-05-12

**Authors:** Monica S. Ruiz, Allison O’Rourke, Sean T. Allen

**Affiliations:** Milken Institute School of Public Health, George Washington University, 950 New Hampshire Ave, Suite 300, Washington, DC 20052 USA

**Keywords:** HIV, PWID, Population estimation, Capture-recapture

## Abstract

No current estimates exist for the size of the population of people who inject drugs (PWID) in the District of Columbia (DC). The WHO/UNAIDS Guidelines on Estimating the Size of Populations Most at Risk to HIV was used as the methodological framework to estimate the DC PWID population. The capture phase recruited harm reduction agency clients; the recapture phase recruited community-based PWID. The 951 participants were predominantly Black (83.9 %), male (69.8 %), and 40+ years of age (68.2 %). Approximately 50.3 % reported injecting drugs in the past 30 days. We estimate approximately 8829 (95 % CI 4899 and 12,759) PWID in DC. When adjusted for possible missed sub-populations of PWID, the estimate increases to 12,000; thus, the original estimate of approximately 9000 should be viewed in the context of the 95 % confidence interval. These evidence-based estimations should be used to determine program delivery needs and resource allocation for PWID in Washington, DC.

## Introduction

Since the identification of the HIV/AIDS epidemic in the District of Columbia (DC), people who inject drugs (PWID) have borne a disproportionate burden of infection risk. The District has seen a 42 % reduction in new cases of HIV among all risk groups between 2008 and 2012 [1]; injection drug use remains the third leading mode of transmission overall and PWID are the third largest group of people living with HIV in DC [1]. Hepatitis C infection (HCV) is also an issue: between 2008 and 2012, 15,915 new cases of chronic HCV were diagnosed among DC residents. In the 2010 National Health and Behavior Survey (NHBS) report on PWID in DC, 90 % of PWID participants indicated they were HCV positive [2].

Existing literature on the size of the PWID population in DC incorporates the states (or portions of the states) surrounding the District to create a “metropolitan area” estimate. For example, in a 2004 article published by Friedman et al., the population estimate for DC also included Maryland, Virginia, and West Virginia and ranged from 5500 to 54,000 [3]. With this estimate including multiple states and the range being so large, it is hard to determine how much of the PWID population actually lives in the District versus elsewhere. This limitation is problematic for organizations serving PWID in DC because they do not know the true size of the population they serve.

Health departments and community organizations need accurate estimates of the size of their target population in order to adequately allocate resources and ensure sufficient service provision. Accurate population size estimates are also useful for mathematical modeling of epidemic impact of prevention efforts. Capture and recapture methods for population estimation have been used to estimate the size of human populations (such as PWID) that are socially marginalized and often hard to quantify [4–6]. These studies often base their estimates on community datasets such as arrests, substance use treatment, or community based service records. However, these types of source data are often incomplete due to fringe populations’ lack of trust in accessing services unless they are allowed to do so anonymously. As a result, it is difficult to find two data sources that can be accurately matched and that represent the entirety of the population.

At present, DC’s health department has been operating HIV prevention programs for PWID (such as syringe exchange services) without an evidence-based estimate of the population size. Implementing a population estimation study in DC has the potential to offer considerable insights into the efficacy and reach of syringe exchange program coverage to PWID. We conducted a capture-recapture study to develop an accurate estimate of the number of PWID in DC using a combination of sources, including data from PWID who are engaging with harm reduction service providers as well as PWID who are not.

## Methods

The capture-recapture study was conducted between March and April 2014 in accordance with the WHO/UNAIDS Guidelines on Estimating the Size of Populations Most at Risk to HIV [10]. We partnered with two local harm reduction service providers who have been engaged in providing services to DC residents since the inception of the DC Department of Health’s needle exchange network (DC NEX) in 2008. Together, these two organizations accounted for 98.2 % of the clean injection equipment (needles and syringes) provided through the DC NEX in fiscal year 2013. Both organizations provide mobile syringe exchange and one organization provides sterile syringe delivery throughout the city. Both organizations serve clients in all eight of The District’s wards.

Two 14-day periods of data collection—the capture phase and the recapture phase—were defined a priori. The capture phase focused on reaching PWID presenting for services at either of the two harm reduction organizations. The recapture phase focused on reaching PWID in the community who are not engaged with services. In the capture phase, recruitment of study participants occurred during routine mobile harm reduction outreach. All individuals presenting for services at mobile syringe exchange locations or those requesting syringe delivery were given the opportunity to participate. In the recapture phase, community outreach workers approached individuals in community locations (e.g., parks, local hangouts, etc.) that were not connected to or associated with any of the formal syringe exchange services. Additionally, recapture participants were recruited through secondary exchange networks, meaning that they obtained their clean paraphernalia through other PWID who themselves engaged with syringe exchange providers and perform bulk distribution of clean needles to others. By focusing our recapture recruitment on community locations and secondary networks, we were able to access populations of PWID who do not wish or who cannot engage directly with formal syringe exchange services.

During both study phases, every individual who was approached received a verbal description of the study and was given the chance to ask questions. If they verbally consented to participate, they completed an anonymous one-page survey asking questions about individuals’ demographic characteristics, current substance use, and methods of obtaining clean injection equipment. Small tokens of appreciation (toiletries kits or new socks) were given to each individual for their time in completing the survey. No personally identifying information was collected from participants at any time during the study.

Each token was labeled with a project logo sticker to allow for easy identification of project participation. Interviewers asked participants if they had already received one of these tokens before with the project logo sticker. Those indicating that they had received a token were categorized as “recounts”, meaning that they had been seen by a study interviewer on more than one occasion (e.g., initially during the capture phase and again during the recapture phase).

The study methodology was executed in accordance with the WHO/UNAIDS Guidelines on Estimating the Size of Populations Most at Risk to HIV [7]. The mathematical equation used to estimate the population size and its 95 % confidence interval are outlined in the WHO/UNAIDS Guidelines mentioned before. In these equations, necessary counts include the number of unique PWID identified during the capture phase, the number of unique PWID in the recapture phase, and the number of individuals identified during the recapture phase who were also interviewed during the capture phase.

All completed survey data were entered into a Microsoft Access database and a 10 % random sample was double checked for errors. *χ*^2^ analyses were completed to determine if differences existed between the capture and recapture period PWID on behavioral, syringe access, and substance use measures. In cases where expected cell counts were less than 5 for 25 % or more of the cells, Fisher’s exact test was used. Population estimate mathematical calculations were completed using Microsoft Excel while all *χ*^2^ analyses were completed using SAS 9.3.

## Results

A total of 951 surveys (244 at capture, 707 at recapture) were completed during the study. The majority of the sample was 40 years of age or older (68.2 %), male (69.8 %), non-Hispanic (76.1 %), African American/Black (83.9 %), and residents of DC (90.9 %). Fifty-three percent of the sample reported having injected drugs in the 30 days prior to the survey. This number was significantly different (*P* < 0.001) between the capture and recapture phase, with 73.4 % of participant indicating injection drug use in the capture population versus 49.9 % in the recapture population. This difference was not surprising given that the capture phase focused recruitment at local harm reduction organizations while the recapture phase focused on finding PWID out in the community. There were no other significant demographic differences observed between the populations sampled during the capture compared to the recapture phase.

Residence in the DC was determined by participant reported zip code or state of residence. Overall, DC has 48 residential zip codes; there were 31 unique DC zip codes reported by participants who indicated IDU in the last 30 days. These zip codes represent all eight wards in the District. Data pertaining to drug use behaviors of those who indicated recent injection (overall and by study phase) and residence in the DC are presented in Table 1. Overall, PWID reported having initiated their drug use at young ages, with 62.8 % reporting initiating injection drug use behavior before the age of 20 and 20.5 % initiating between the ages of 20 and 29. A significant difference was found in the 40 and older age of injection drug use behavior initiation category between the capture and recapture PWID, with significantly more PWID in the capture phase reporting injection initiation during this time frame (5.0 vs. 1.2 %, *P* < .05). When participants were asked to indicate their injection drug of choice, heroin alone was the most often cited drug (39.3 %), followed by an equal preference for using either heroin or cocaine/speedball (19.0 %), and then cocaine alone (12.6 %). However, significant differences were found in drug of choice between the capture and recapture PWID categories for cocaine, heroin, and only hormones/silicone. Significantly more recapture PWID reported cocaine as their injection drug of choice (2.9 vs. 16.6 %, *P* < .05); however, significantly more capture PWID reported use of heroin alone (45.7 vs. 36.7 %, *P* < .05), or only hormones/silicone (1.4 vs. 0 %, *P* < .05) as their injection drug of choice.

**Table 1:** Substance use behaviors of participants reporting injection drug use within 30 days of survey and DC as residence (N = 478)

	All	Capture	Recapture
	(n = 478)	(n = 140)	(n = 338)
Age initiated injection drug use			
<20	300 (62.8 %)	80 (57.1 %)	220 (65.1 %)
20–29	98 (20.5 %)	29 (20.7 %)	69 (20.4 %)
30–39	30 (6.3 %)	13 (9.3 %)	17 (5.0 %)
40+	11 (2.3 %)	7 (5.0 %)	4 (1.2 %)^a^
Missing	39 (8.2 %)	11 (7.9 %)	28 (8.3 %)
Injection drug of choice			
Cocaine	60 (12.6 %)	4 (2.9 %)	56 (16.6 %)^a^
Heroin	188 (39.3 %)	64 (45.7 %)	124 (36.7 %)^a^
Heroin or cocaine/speedball	91 (19.0 %)	22 (15.7 %)	69 (20.4 %)
Only hormones/silicone	2 (0.4 %)	2 (1.4 %)	0^a^
PCP	14 (2.9 %)	6 (4.3 %)	8 (2.4 %)
Other	84 (17.6 %)	30 (21.4 %)	54 (16.0 %)
Missing	39 (8.2 %)	12 (8.6 %)	27 (8.0 %)
Methods used to obtain clean syringes in last 30 days			
NEX	267 (55.9 %)	100 (71.4 %)	167 (49.4 %)
Bought at pharmacy	15 (3.1 %)	12 (8.6 %)	3 (0.9 %)^a^
Bought from person	83 (17.4 %)	19 (13.6 %)	64 (18.9 %)^a^
Ordered online	3 (0.6 %)	3 (2.1 %)	0
Secondary exchange	124 (25.9 %)	53 (37.9 %)	71 (21.0 %)
Other	18 (3.8 %)	12 (8.6 %)	6 (1.8 %)^a^
Missing	126 (26.4 %)	13 (9.3 %)	113 (33.4 %)
Preferred method for obtaining clean syringes			
NEX	228 (47.7 %)	91 (65.0 %)	137 (40.5 %)^a^
Buy at store or online	5 (1.0 %)	2 (1.4 %)	3 (0.9 %)
Friend or secondary exchange	70 (14.6 %)	17 (12.1 %)	53 (15.7 %)
NEX or friend/secondary	31 (6.5 %)	10 (7.1 %)	21 (6.2 %)
Missing	144 (30.1 %)	20 (14.3 %)	124 (36.7 %)
Drug(s) used in the last 90 days			
Alcohol	264 (55.2 %)	81 (57.9 %)	183 (54.1 %)
Amphetamines	12 (2.5 %)	10 (7.1 %)	2 (0.6 %)^a^
Cocaine or crack	198 (41.4 %)	49 (35.0 %)	149 (44.1 %)^a^
Hallucinogens	11 (2.3 %)	7 (5.0 %)	4 (1.2 %)^a^
Heroin	290 (60.7 %)	107 (76.4 %)	183 (54.1 %)^a^
Hormones/silicone	8 (1.7 %)	5 (3.6 %)	3 (0.9 %)
Inhalants	2 (0.4 %)	0	2 (0.6 %)
Marijuana	144 (30.1 %)	31 (22.1 %)	113 (33.4 %)^a^
Prescription drugs	73 (15.3 %)	10 (7.1 %)	63 (18.6 %)^a^
Other	12 (2.5 %)	11 (7.9 %)	1 (0.3 %)^a^
Missing	32 (6.7 %)	2 (1.4 %)	30 (8.9 %)

^a^ *χ* ^2^ test significant at *P* < .05 between capture and recapture IDU

When asked to indicate all the methods used to obtain clean injection equipment in the last 30 days overall, the DC NEX was most often reported (55.9 %) followed by secondary exchange (25.9 %), and purchasing them from someone else (13.6 %). Capture PWID were also significantly more likely than recapture PWID to have obtained clean syringes by buying them at a pharmacy (8.6 vs. 0.9 %, *P* < .05) or getting them through other non-NEX sources (8.6 vs. 0.9 %, *P* < .05). Recapture PWID were significantly more likely than capture PWID to have obtained clean syringes by purchasing them from another person (13.6 vs. 18.9 %, *P* < .05). Capture PWID were significantly more likely than recapture PWID to name the NEX as a preferred source for clean syringes (65.0 vs. 40.5 %, *P* < .05).

When participants were asked to indicate what substances they had used the last 90 days, heroin (60.7 %), alcohol (55.2 %), and cocaine (41.4 %) were the three most frequently reported substances overall. However, differences in drugs used were observed for each study phase. Compared to recapture participants, capture PWID were significantly more likely to report using amphetamines (5.6 vs. 0.6 %, *P* < .05), hallucinogens (7.1 vs. 0.6 %, *P* < .05), heroin (76.4 vs. 54.1 %, *P* < .05), and hormones (3.6 vs. 0.9 %, *P* < .05) while recapture PWID were significantly more likely to report using cocaine or crack (35.0 vs. 44.1 %, *P* < .05), marijuana (22.1 vs. 33.4 %, *P* < .05), or prescription drugs (7.1 vs. 18.6 %, *P* < .05).

To calculate the population estimate using the WHO/UNAIDS mathematical formula, we calculated [1] the number of unique PWID interviewed during the capture period, [2] the number of unique PWID interviewed during the recapture period, and [3] the number of PWID contacted at both periods. During the capture period, 244 individuals were interviewed however, only 140 (57.4 %) of these reported injecting during the previous 30 days and DC as their state of residence; of these, 9 individuals were identified as being repeat participants (i.e., they had taken the survey twice during the capture period) and were therefore removed from the total. This resulted in a total of 131 unique PWID identified during the capture period. Of the 707 individuals interviewed during the recapture period, 338 (47.8 %) individuals reported injecting drugs in the previous 30 days. Of these, one survey was removed due to missing data, leaving a final count of 337 unique PWID identified in the recapture period. Of these 337 individuals, 5 individuals indicated that they had completed the survey during both the capture and recapture time periods. Using these numbers in the equation, 131 capture PWID, 337 recapture PWID, and 5 individuals seen at both time periods, we estimate that there are 8829 (95 % CI 4899 and 12,759) PWID in Washington, DC.

## Discussion

Using capture-recapture methodology, we estimate that there are approximately 9000 PWID in the DC; this number represents approximately 1.8 % of the District’s 500,908 persons aged 18 years and older [8]. A more accurate estimate is important to the District’s HIV and HCV prevention and control efforts because it provides the Department of Health and community-based organizations serving the PWID populations with a better idea of the true size of the client-base for harm reduction services. With better understanding of the size of the population needing services, the city can more easily and accurately evaluate how well the DC NEX and other harm reduction organizations are reaching and serving the population.

In an effort to obtain a “wisdom of the crowd” perspective regarding this estimate, we asked our community harm reduction service provider partners about their thoughts regarding the accuracy of the estimate based on their direct experience in serving the PWID community. One of the two organizations believed that our 8829 calculation was an overestimate whereas the other organization believed it to be an underestimate. Nonetheless, both organizations agreed that the true estimate fell within our 95 % confidence interval.

More accurate population estimates allow for appropriate budgeting of resources for the provision of necessary services and other prevention programming. In 2012, the World Health Organization (WHO), United Nations Office on Drug and Crime (UNODC), and UNAIDS released a technical guide for countries to set targets for universal access to HIV prevention, treatment and care for injecting drug users [9]. Among these recommendations was the determination of adequate syringe coverage given the size of the PWID population. According to the guidance, coverage can be classified based on the mean number of syringes distributed per PWID into three categories: low (less than 100), medium (100–200), and high (over 200). Based on our estimations of the PWID population in the District and the number of clean syringes distributed by the DC NEX in fiscal year 2013 (n = 684,000), DC is currently providing 77.5 syringes per PWID (95 % CI 139.6, 53.6), which falls into the “low coverage” category. In order to fall in the middle of the “medium coverage” category, the DC NEX would need to distribute approximately 1.3 million clean syringes (95 % CI: 735,000, 1.9 million), which would mean doubling the current amount of syringe distribution. Achieving the goal of medium syringe coverage has obvious implications for program planning and resource allocation.

Two factors were identified that may have affected the validity of the IDU population estimate presented in this manuscript: the use of a 30-day reporting time period for injection behavior and the ability of this study to access the most hidden PWID, including new/young injectors and middle class injectors. Using a 30-day reporting period for having engaged in injection behavior was done to minimize recall errors. Research has shown that a shorter 30-day window versus a longer 6-month window can result in a 15 % reduction in identified population [10]. If we apply this to our estimate by increasing the number of PWID identified during each phase, our estimate would increase from about 9000 to 12,000 individuals. While this is a substantial increase, it is still within our confidence interval for the 30-day reporting period estimate.

Another factor that may affect the validity of our estimate was the ability of this study to access the most hidden segments of this already hard to reach population, such as the younger or newer PWID or those from higher socioeconomic brackets. We attempted to include all segments of the PWID population, not just those who engage with the DC NEX, by recruiting individuals who obtained clean paraphernalia through secondary exchangers and individuals who only engaged with community outreach workers for harm reduction supplies. While these methods were intended to minimize missed segments of the broader PWID population, it is possible that there are still subpopulations of PWID who are not engaged with any harm reduction services and, therefore, would not be seeing secondary exchangers or community outreach workers. There were also time and financial constraints associated with attempting to reach these subpopulations. Further, given that we only included in our analyses individuals who explicitly indicated that they were DC residents, our estimate does not reflect the individuals who may actually reside in DC but who were not comfortable providing their zip code of residence (11.5 % of the injectors surveyed in our study) or who reside outside the District but who come to the city for services (5 % of the injectors surveyed). Had these uncounted and non-resident individuals been included in the calculations, our population estimate increases to approximately 12,000 injectors (95 % CI 7000, 16,500). Given these limitations and considerations, we recommend that the population estimate of approximately 9000 be viewed in the context of being contained within the 95 % confidence interval, which would accommodate the possible missed populations of PWID who were not accessed.

In examining the population sampled for this study, some significant differences were identified between the capture and recapture groups in terms of their substance use behaviors and harm reduction practices. While heroin was the most often reported injection drug of choice for both groups, there were significantly more cocaine injectors in our recapture group. Given that our recapture group was from the community rather than from syringe exchange sites, it is possible that the sites are not adequately reaching stimulant injectors with harm reduction services. More efforts should be undertaken to better understand the particular needs of stimulant injectors and how harm reduction providers can more successfully engage them.

With regard to harm reduction behaviors, those in the capture group were more likely to indicate more than one method of obtaining clean syringes in the past 30 days compared to those in the recapture group. This finding may indicate that those who engage with the NEX already prioritize the importance of having clean injection equipment. It may also indicate how well harm reduction service providers in the DC NEX emphasize the importance of clean needle and syringe use, as well as educate clients on different options for obtaining clean equipment. Such information and education may not be available to those who choose to not engage with the NEX.

Additionally, we found that while injectors report having initiated injection practices at younger ages (e.g., in their 20s), they are engaging in needle exchange services when they are much older (e.g., in their 40s). While this finding may be an artifact of simply having an older PWID population in the District, it also points to the possibility that young injectors in DC and adjacent areas who are at the start of their substance use careers are not aware of the harm reduction services that are available to them and, therefore, are not accessing services. More research is needed to better understand the diversity of the substance using population in the DC area so that efforts can be made to reach and engage younger populations with harm reduction information and services, including overdose prevention.

One of the greatest strengths of this study is that we were able to engage local harm reduction service providers as collaborators. Their expertise and trust within the PWID population allowed us access to a population that is often wary of outsiders, particularly researchers. Also, we were able to work with our community partners in a manner that did not disrupt their normal routines for service provision to PWID clients. This successful collaboration demonstrates that community based organizations, even those with little experience with research, can be active and valuable partners as long as researchers respect their needs and do not jeopardize or detract from their mission of service provision. When the terms of engagement are based in respect, the collaboration between research and practice can be mutually beneficial.

This study provides an evidence-based estimation of the number of PWID in DC. These data are useful in that they provide better information for harm reduction service providers to estimate program delivery needs. With more accurate population estimates guiding the resource allocation and program implementation, DC’s harm reduction providers will have greater capacity to meet the needs of the approximately 9000 PWID at disproportionate risk for HIV and HCV.
